# Effect of health insurance coverage on labor allocation: evidence from US farm households

**DOI:** 10.1186/s13561-014-0019-1

**Published:** 2014-10-29

**Authors:** Jeremy M D’Antoni, Ashok K Mishra, Aditya R Khanal

**Affiliations:** Economist at Economic Research Service in the United States Department of Agriculture, Washington DC, USA; Department of Agricultural Economics and Agribusiness, Louisiana State University AgCenter and Louisiana State University, Baton Rouge, LA 70803 USA

**Keywords:** C34, I13, J12, J22, J38, J43, Q12, Q18, Health insurance coverage, Endogeneity, Copula, Off-farm labor supply, Dependence, Bivariate tobit, Coupled farm programs payments, Decoupled farm program payments

## Abstract

In the past three decades, farm families have relied on government payments and off-farm income to reduce income risk and increase total household income. Many studies have analyzed the role of government payments; however, little is known about the impact of health insurance coverage on labor allocation. This study builds on previous literature by using copulas to test for dependence in the labor allocation, addressing the importance of fringe benefits to the farm household, and determining how these considerations affect our knowledge of the impact of fringe benefits on off-farm labor. The results indicate that the off-farm hours worked by the operator and spouse are jointly determined; health insurance coverage is an endogenous variable. Using the predicted probability of insurance coverage and joint estimation techniques, we find a positive and highly significant relationship with the hours worked off-farm. Further, we find that both coupled and decoupled payments are negatively correlated with the hours worked off-farm.

## Background

During the past three decades, self-employed farm households have engaged in dual employment—farm and off-farm work. Dual employment has provided a critical income source to a majority of self-employed farm households not only in the U.S. and Western European countries, but in developing economies as well (e.g., [1-6]). Economists have investigated several issues—particularly public policy, education, and wealth—that impact the dual employment decisions of self-employed farm operators and/or spouses.

Most surveyed farm families claim that off-farm work provides extra, much-needed income to support family expenditures^a^ [2]. Yet, surveys conducted by the USDA indicate reasons unrelated to the farm business, from buying groceries to funding retirement accounts as reasons for off-farm work [7]. Anecdotal evidence suggests that many self-employed business owners and/or their family members work off-farm to provide fringe benefits. Fringe benefits are viewed as a component of off-farm wage. Jensen and Salant [8] demonstrate the positive correlation between fringe benefits and the number of hours farmers are employed off the farm. However, Jensen and Salant’s data is mainly local and small in scope (800 farms in Mississippi and Tennessee). Additionally, this research was conducted prior to both the decoupling of government payments and significant increases in healthcare costs.

**Figure 1:**
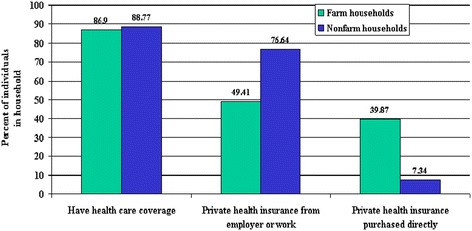
**Heath care coverage and source of health care coverage (National Health Interview Survey, 1997–2004).**

Figure 1 shows that only 50 percent of individuals from farm families have employer-provided health insurance. Given the extensive engagement of farm-operator households in the nonfarm economy, it is not surprising that the most common source of health insurance for members of farm households is employment-based. This is in stark contrast to nearly 75% of the non-farm sector having private health insurance from employers. Even more staggering, approximately 40 percent of individuals from farm households—compared to the 7 percent of individuals in all other U.S. households—purchase health insurance directly from a vendor. Without accounting for additional income, working off-farm can substantially decrease financial stress for farm households by providing fringe benefits (health insurance in particular). Programs such as public healthcare might benefit farm households by decreasing the need to personally fund these benefits, work fewer hours off-farm, and work more hours on the farm—especially if there are non-pecuniary benefits to farm labor.

Until now, these considerations have been difficult to model due to a lack of data. In particular, the literature has been all but silent on the probability of getting employer-provided health insurance from an off-farm job and, if received, the subsequent impact on off-farm work.

During the past two decades, considerable research has focused on agricultural policy and its unintended consequences on factor markets. Among this research are analyses of the labor allocation decisions of farm families. Factors such as non-farm, economic growth and farm program payments have been tied to the migration of farm operators and spouses to off-farm work. Often overlooked is the role of fringe benefits, like health insurance and retirement plans, in drawing farm households to work off-farm. D’Antoni and Mishra [9] demonstrate that the welfare lost by farm households from a reduction in decoupled government payments may be overstated when fringe benefits are excluded from the estimation of off-farm labor supply.

An issue that has garnered attention in the literature is the interdependence of farm operators and spouses in off-farm work. Most dual employment studies begin by using a theoretical household model that posits jointness in farm couples’ decisions to participate in off-farm work. Researchers then estimate bivariate probit/Tobit models for off-farm labor of the operator and spouse. They then test for jointness *post hoc* via hypothesis testing for the correlation parameter (rho) between the error terms of the simultaneous equations. We find, however, that copulas provide a more efficient and consistent procedure for testing dependence early in the investigative process. The results of this test can then be used to guide empirical methods rather than the status quo of simple verification.

Therefore, the objective of this study is threefold. First, we utilize copulas to measure the dependence between married farm couples’ off-farm labor supply. Unlike the previous studies, copulas provide a consistent procedure for testing dependence and guiding the choice of empirical model. Secondly, we test for endogeneity of health insurance coverage and determine the impact of this fringe benefit on the off-farm labor supply of farm operators and spouses. Thirdly, we assess the impact of reduced decoupled payments^b^ on the off-farm labor supply of farm operators and spouses. The data used in this research is from the 2006–2008 Agricultural Resource Management Survey (ARMS). The results of our copula test indicate that the labor supply decisions of farm couples are best modeled jointly. Using a bivariate Tobit model, we find health insurance coverage has a highly significant and positive impact on the off-farm labor supply of operators and spouses. Finally, meaningful evidence indicates that a reduction in decoupled payments would increase the hours worked off-farm by the operator and spouse.

### Health insurance for self-employed farmers in the United States

Unlike health care system in Western Europe, where government plays a significant role in the healthcare sector, the health care system in the United States is employer-sponsored health insurance, which is offered to employees as part of a compensation package. Such a system can present challenges to self-employed individuals, such as farmers and ranchers, and their families. Although most farmers are self-employed, the share of farm operator household members that have no form of health insurance is about the same as that of the overall U.S. population (13 and 16 percent, respectively). A farm business does not generally offer employment-based health insurance, but other factors help to equalize insurance coverage. Farm operator households spend much more on health care than the average of all U.S. households, largely due to their greater reliance on direct-purchase private health insurance. For example, in 2007, farm households spent, on average, $5,200 for both health insurance premiums and out-of-pocket health costs. For the 40 percent of farm operator households for whom farming is their primary occupation, health expenses are even higher, averaging nearly $6,000 in 2007. Primary-occupation farm households have higher health expenses than other farm households, even when they include household members working off the farm. For the primary-occupation households that rely solely on direct purchase health insurance, total out-of-pocket health expenses averaged nearly $10,000 in 2007 [10].

However, in the majority of farm operator households, the operator or spouse is also employed off the farm, so the most common source of health insurance for members of farm households is employment based. In addition, having health insurance increases with a person's age and income. Farm operator households are more than three times as likely as other household types to be headed by an individual over age 65. Farm operator households also have higher incomes and net worth, on average, than the general U.S. population, but much of their net worth is tied up in their farming business and farm income can vary widely from year to year. Given that farm operator households have relatively high net worth and tend to rely on family labor to operate the farm business, adequate health insurance coverage for household members may be as important to the financial security of the farm business as it is to the health of the family.

## Methods

The following theoretical model of the farm household illustrates the operator/spouse dependence in off-farm hours worked [3,11]. The farm household is comprised by the farm operator (*O*) and spouse (*S*) and follows a utility maximization framework which assumes that utility (*U*) is a function of leisure (*L*) and income (*I*).

1 $$ Max\ U=U\left({L}^o,{L}^s,I\right) $$

$$ s.t. $$

2 $$ {L}^o+{F}^o+{E}^o\left({E}^s\right)-T=0 $$

3 $$ {L}^s+{F}^s+{E}^s\left({E}^o\right)-T=0 $$

4 $$ {w}_E^o{E}^o\left({E}^s\right)+{w}_E^s{E}^s\left({E}^o\right)+{\pi}_F + V-I=0 $$

5 $$ {L}^o,{F}^o,{E}^o\left({E}^s\right)\ge 0\ {L}^s,{F}^s,{E}^s\left({E}^o\right)\ge 0 $$

Utility maximization in equation (1) is subject to the total available hours (*T*) allocable to leisure (*L*), farm labor (*F*), and off-farm labor (*E*) of the operator (Equation 2) and spouse (Equation 3), the full income constraint (Equation 4), and non-negativity constraints (Equation 5). Notice that the off-farm hours of the operator (*E*^*o*^) are a function of the off-farm hours worked by the spouse (*E*^*s*^). This explicitly demonstrates the jointness in off-farm labor allocation. The full income constraint is defined as the sum of income from the operator’s off-farm labor (${w}_E^o{E}^o\left({E}^s\right)$), spouse’s off-farm labor (${w}_E^s{E}^s\left({E}^o\right)$), farm profits (*π*_*F*_), and other household non-labor income (*V*) minus the total income (*I*). Farm profits are further defined as the value of farm production minus the input costs. Specifically,

6 $$ {\pi}_F={P}_ff\left({F}^o,{F}^s,{X}_f\right) - v{X}_f $$

The Lagrangian (L) can be constructed for the outlined maximization problem with the following first order conditions for off-farm labor:

7 $$ U\kern0.5em \left({L}^o,\kern0.5em {L}^s,\kern0.5em I\right)\kern0.5em +\kern0.5em {\lambda}_1\left({L}^o+{F}^o+{E}^o\left({E}^s\right)-T\right) + {\lambda}_2\kern0.5em \left({L}^s+{F}^s+{E}^s\left({E}^o\right)-T\right) + \delta \left({w}_E^o{E}^o\left({E}^s\right)+{w}_E^s{E}^s\left({E}^o\right)+{\pi}_F + V-I\right) $$

8 $$ \frac{\partial \mathrm{L}}{\partial {E}^o}\Rightarrow MR{S}_{L,I}^o-{w}_E^o = \left(\frac{\partial {E}^s}{\partial {E}^o}\right)\left\{{w}_E^s-MR{S}_{L,I}^s\right\} $$

9 $$ \frac{\partial \mathrm{L}}{\partial {E}^s}\Rightarrow MR{S}_{L,I}^s-{w}_E^s = \left(\frac{\partial {E}^o}{\partial {E}^s}\right)\left\{{w}_E^o-MR{S}_{L,I}^o\right\} $$

Per the cross-partial derivative in equations (8) and (9), if the off-farm labor allocation decision of the spouse is independent of the operator, then $\frac{\partial {E}^s}{\partial {E}^o}=\frac{\partial {E}^o}{\partial {E}^s}$ = 0. For the operator, this implies that utility is maximized where the marginal rate of substitution ($MR{S}_{L,I}^o$) between leisure and income is exactly equal to the off-farm wage. According to Ahearn, El-Osta, and Dewbre [3], there is conflicting empirical evidence supporting the validity of independence. This will be formally tested using the copula methods described by [9].

If we allow for dependence in our theoretical model and expand our concept of the “wage” earned from off-work, we can say the full off-farm wage is a function of both the hourly wage (*w*) and fringe benefits (*f*_*b*_). Therefore, ${w}_E^o$ and ${w}_E^s$ can be further defined ${\overline{w}}_E^o\left({w}_E^o,\ {f}_b\right)$ and ${\overline{w}}_E^s\left({w}_E^s,\ {f}_b\right).$ By rearranging the first order conditions in equations (8) and (9) and including our newly defined terms for off-farm wage, we can see more clearly the impact of health insurance on the off-farm labor allocation decisions of farm families^c^.

10 $$ \frac{\partial \mathrm{L}}{\partial {E}^o}\Rightarrow MR{S}_{L,I}^o + \left(\frac{\partial {E}^s}{\partial {E}^o}\right)MR{S}_{L,I}^s = {\overline{w}}_E^o\left({w}_E^o,\ {f}_b\right) + \left(\frac{\partial {E}^s}{\partial {E}^o}\right){\overline{w}}_E^s\left({w}_E^s,\ {f}_b\right) $$

11 $$ \frac{\partial \mathrm{L}}{\partial {E}^s}\Rightarrow MR{S}_{L,I}^s + \left(\frac{\partial {E}^o}{\partial {E}^s}\right)MR{S}_{L,I}^o = {\overline{w}}_E^s\left({w}_E^s,\ {f}_b\right) + \left(\frac{\partial {E}^o}{\partial {E}^s}\right){\overline{w}}_E^o\left({w}_E^o,\ {f}_b\right) $$

The total wage is non-decreasing in ${w}_E^o,\ {w}_E^s,$ and *f*_*b*_ (holding all else constant). For example, an increase in health insurance benefits received off-farm will increase *f*_*b*_. In equation (11), an increase in *f*_*b*_ will result in an increase in ${\overline{w}}_E^s\left({w}_E^s,\ {f}_b\right)$. If labor decisions are made jointly, then $\left(\frac{\partial {E}^o}{\partial {E}^s}\right){\overline{w}}_E^o\left({w}_E^o,\ {f}_b\right)$ will either increase or decrease depending on the direction of dependence.

Based on the empirical results of D’Antoni and Mishra ([9] using copulas and the post-hoc tests of Ahearn et al. (1996), we assume $\left(\frac{\partial {E}^o}{\partial {E}^s}\right)>0$ and increasing fringe benefits will increase the total off-farm wage earned by the spouse; resulting in an increase in the right hand side of the equality in equation (11) implying greater substitution of leisure for income and greater hours worked off-farm. It’s important to note that the discussed changes in fringe benefits under joint labor decisions will alter the off-farm labor supply of both the operator and spouse.

### Off-farm labor supply: an empirical model

A seemingly unrelated regression model can be adapted such that a Tobit model can be used rather than simple regression [12]. Specifically,

12 $$ {Y}_1^{*}={X}_i{\beta}_i+{\delta}_O{H}_O+{\varepsilon}_1 $$

13 $$ {Y}_1=\left\{\begin{array}{c}\hfill {Y}_1^{*}\  if\ {Y}_1^{*}>0\hfill \\ {}\hfill 0\  Otherwise\ \hfill \end{array}\right. $$

14 $$ {Y}_2^{*}={X}_k{\beta}_k+{\delta}_S{H}_S+{\varepsilon}_2 $$

15 $$ {Y}_2=\left\{\begin{array}{c}\hfill {Y}_2^{*}\  if\ {Y}_2^{*}>0\hfill \\ {}\hfill 0\  Otherwise\ \hfill \end{array}\right. $$

Equations (12) and (13) represent off-farm labor supply equations for the operator and spouse. ${Y}_1^{*}$ and ${Y}_2^{*}$ are the untruncated latent variables allowing for theoretically negative values which represent hours worked off-farm by the operator and spouse, respectively.*Y*_*1*_ and *Y*_*2*_ are the left-censored dependent variables for off-farm hours worked by the operator and spouse, respectively. Vectors *X*_*i*_ and *X*_*k*_ are explanatory variables. We separately denote the explanatory variables, insurance coverage variable for the operator (*H*_*O*_) and spouse (*H*_*S*_), and error terms for operator (*ε*_1_) and spouse (*ε*_2_). These disturbances are jointly normally distributed with variances ${\sigma}_1^2$ and ${\sigma}_2^2$ where ${\varepsilon}_1,\ {\varepsilon}_2\sim N\left(0,\ 0,\ {\sigma}_1^2,\ {\sigma}_2^2,\rho \right)$ and the covariance is given by *σ*_1,2_ = *ρσ*_1_*σ*_2_.

The results from this model are not directly interpretable as the marginal effect of the independent variable on the dependent variable; therefore, we further calculate the marginal effects. According to Greene [13], the marginal effects in a bivariate Tobit context can be calculated in the same manner as a univariate model:

16 $$ \frac{\partial E(Y)}{\partial X}=\Phi \left(X\beta /\sigma \right)\beta $$

where Φ(*Xβ*/*σ*) is the cumulative normal distribution function.

The explanatory variables (*H*_*O*_) and (*H*_*S*_) are dummy variables representing whether the operator or spouse obtains health insurance coverage from off-farm sources. In prior studies, researchers [2,5,14-17] began by implementing a theoretical household model where the labor allocation decision is jointly determined. When joint estimation methods were used, bivariate probit/Tobit models were estimated. A correlation parameter (*rho),* that measures the dependence between the error terms of two simultaneous off-farm labor functions, was then tested against the null hypothesis of *rho* equals zero. The empirical evidence of dependence is inconclusive in these studies. Furthermore, this method assessed the appropriateness of the empirical model for the theory *post hoc*. In these studies, testing the dependence between operator and spouse labor decisions has been approached more from the perspective of testing “goodness of fit” rather than as a useful tool for guiding model selection. Following D’Antoni and Mishra [9], we use copulas to test whether $\frac{\partial {E}^s}{\partial {E}^o}$ and $\frac{\partial {E}^o}{\partial {E}^s}$ are significantly different from zero and to determine whether dependence is positive or negative.

Based on the literature, we suspect that health insurance coverage is determined jointly with the number of off-farm hours worked. The Smith-Blundell test is used to test for endogeneity because the structural model is a Tobit [18]. Under this test, the null hypothesis states that all variables are exogenous while the alternative hypothesis states that insurance coverage is a linear projection of a set of instruments. The residuals from this first stage regression are added to the model. If the null is not rejected, then these residuals have no explanatory power. However, for both the operator and spouse equations the null hypothesis is rejected.

### Endogeneity of health insurance coverage

To address endogeneity of the health insurance variable in our model, we use the predicted probability of insurance coverage for the operator and spouse. Following the results of our copula test, we model the predicted probability of insurance coverage jointly using the bivariate probit model. According to Greene [13] and Ahearn, El-Osta, and Dewbre [3]

17 $$ {y}_1^{*}=\left\{\begin{array}{c}\hfill {\beta}_1^{\hbox{'}}{X}_1+{\varepsilon}_1\  if\ {y}_1=1\hfill \\ {}\hfill 0\  if\ {y}_1=0\ \hfill \end{array}\right. $$

18 $$ {y}_2^{*}=\left\{\begin{array}{c}\hfill {\beta}_2^{\hbox{'}}{X}_2+{\varepsilon}_2\  if\ {y}_2=1\hfill \\ {}\hfill 0\  if\ {y}_2=0\hfill \end{array}\right. $$

19 $$ E\left[{\varepsilon}_1\Big|{X}_1,\ {X}_2\right]=E\left[{\varepsilon}_2\Big|{X}_1,\ {X}_2\right]=0 $$

20 $$ var\left[{\varepsilon}_1\Big|{X}_1,\ {X}_2\right]=var\left[{\varepsilon}_2\Big|{X}_1,\ {X}_2\right]=1 $$

21 $$ E\left[{\varepsilon}_1,\ {\varepsilon}_2\Big|{X}_1,\ {X}_2\right]=\theta $$

where *y*_1_ and *y*_2_ are binary variables indicating health insurance coverage from off-farm work for the operator and spouse, *X*_1_ and *X*_2_ are vectors of exogenous variables, *β*_1_ and *β*_2_ are vectors of estimated parameters, *ε*_1_ and *ε*_2_ are error terms, and *θ* is the coefficient of correlation between the error terms.

When specifying the equations in the bivariate probit model, there must be at least one variable that is highly correlated with the dependent variable in equations (17) and (18) but uncorrelated with the dependent variables in (12) and (14). There are two exogenous instruments used in these equations. The first variable indicates personal expenditure on insurance, health, and retirement benefits. The second variable indicates expenditure on fringe benefits for hired workers. The latter variable is expected to be negatively correlated with operator/spouse insurance coverage from off-farm work. If a farm household is going to pay for benefits to cover its hired workers, then it is more likely to cover itself as well. Personal expenditure on insurance, health, and retirement benefits is indeterminate in sign. It can be argued that farm households expending personal funds on these benefits are less likely to be covered by other sources; they likely pay these expenses out of necessity. Conversely, it can be argued that those paying for these expenses are more concerned about being fully insured and financially secure; therefore, they seek out off-farm employment providing these benefits as well.

In addition to these exogenous instruments, we regress operator and spouse insurance coverage on age, age squared, education, and household size. The results from this regression are found in Table 1. Notice the explanatory variables are all significant at 1%. We further test for instrument strength using a joint F-test for the operator and spouse equations. The F-test value for each equation was large and significant at 1%; therefore, we can reject the null that all parameters are jointly equal to zero and conclude that at least one of our instruments in each equation is not weak.

**Table 1:** **Bivariate probit results for the predicted value of insurance coverage**

**Variable**	**Operator coefficient (Std Error)**	**Spouse coefficient (Std Error)**
Age	0.0735***	0.0708***
	(0.0120)	(0.0119)
Age Squared	−0.0008***	−0.0008***
	(0.0001)	(0.0001)
Education	0.0714***	0.1411***
	(0.0077)	(0.0072)
Household Size	−0.0414***	−0.0747***
	(0.0114)	(0.0110)
Personal Insurance Policy	0.1736***	0.2188***
	(0.0293)	(0.0267)
Fringe Benefit to Hired Labor	−1.0846***	−0.3356***
	(0.0320)	(0.0260)
Constant	−2.8621***	−3.6140***
	(0.3175)	(0.2972)
**N**	11,262	
*χ* ^*2*^	2,118.71***	

***, **, and * indicate significance at the 1%, 5%, and 10% levels, respectively.

From these results, we calculate the predicted probability of the operator having health insurance from off-farm sources holding the spouse’s equation constant and vice versa. These predicted values will then be used in our structural model outlined by equations (12) - (14). These equations can be rewritten with the predicted values notated ${\widehat{H}}_O$ and ${\widehat{H}}_S$ as:

22 $$ {Y}_1^{*}={X}_i{\beta}_i+{\delta}_O{\widehat{H}}_O+{\varepsilon}_1 $$

23 $$ {Y}_1=\left\{\begin{array}{c}\hfill {Y}_1^{*}\kern1.75em  if\ {Y}_1^{*}>0\hfill \\ {}\hfill 0\kern1.25em  Otherwise\ \hfill \end{array}\right. $$

24 $$ {Y}_2^{*}={X}_k{\beta}_k+{\delta}_S{\hat{H}}_S+{\varepsilon}_2 $$

25 $$ {Y}_2=\left\{\begin{array}{c}\hfill {Y}_2^{*}\kern1.75em  if\ {Y}_2^{*}>0\hfill \\ {}\hfill 0\kern1.75em  Otherwise\ \hfill \end{array}\right. $$

This bivariate Tobit model will be estimated via maximum likelihood. Due to the use of predicted values and complex survey design, standard errors were calculated using bootstrapping techniques. Following Cameron and Trivedi [19], 500 iterations were used in our calculation of standard errors.

### Data

This research utilizes Agricultural Resource Management Survey (ARMS) data from 2006 to 2008. The ARMS is conducted annually by the Economic Research Service and the National Agricultural Statistics Service (for more detail, see http://www.ers.usda.gov/data-products/arms-farm-financial-and-crop-production-practices.aspx#.u_swgfldwso). The survey collects data to measure the financial condition (farm income, expenses, assets, and debts) and operating characteristics of farm businesses, the cost of producing agricultural commodities, and the well-being of farm operator households. The target population of the survey is operators associated with farm businesses representing agricultural production in the 48 contiguous states. Each survey is collected from a single, senior farm operator who makes most of the day-to-day management decisions. We also collected wage data from the Bureau of Labor Statistics. By state and year, we calculate the weighted average wage earned from off-farm work, weighting according to the portion of the state population employed in each sector.

The list of variables, with summary statistics, used in our labor supply model can be found in Table 2. We limited our study to farm households where either the farm operator or spouse is under the age of 65, resulting in a sample size of 11,262 farm households. This excludes all households that are fully covered by Medicare. We also exclude all households that did not respond to hours worked off-farm or reported hours per week worked on or off-farm greater than 140. This applies to farms that reported 140 worked at either location separately or additively. In other words, any operator or spouse responding that they on average sleep fewer than four hours per night is assumed to have incorrectly completed the survey and are dropped.

**Table 2:** **Summary statistics and variables used in the study**

**Variables**	**Description**	**Mean**	**Std Dev**
*Operator_off-farm*	Off-Farm hours per week	13.99	(20.14)
*Spouse_off-farm*	Off-Farm hours per week	22.58	(19.59)
*Op_Age*	Age in years	51.74	(9.61)
*Sp_Age*	Age in years	49.34	(9.15)
*Op_Educ*	Total years of education	13.62	(1.85)
*Sp_Educ*	Total years of education	13.94	(1.91)
*Op_Miles*	Miles from off-farm job	5.79	(29.64)
*Sp_Miles*	Miles from off-farm job	9.96	(97.84)
*Op_Hinsurance*	1 if the operator has health insurance through off-farm work; 0 Otherwise	0.21	(0.41)
*Sp_Hinsurance*	1 if the spouse has health insurance through off-farm work; 0 Otherwise	0.30	(0.46)
*Of_Wage*	Hourly off-farm wage rate	21.11	(1.80)
*Decoupled_Payments*	Annual payments in $1,000	9.87	(24.33)
*Coupled_Payments*	Annual payments in $1,000	10.81	(36.23)
*Farm_Sales*	Total value of farm sales in $1,000	370.42	(2423.67)
*Household_size*	Number of members of household	3.17	(1.43)
*F_Dairy*	1 if the farm specializes in dairy farming; 0 Otherwise	0.12	(0.32)
*R_Heartland*	1 if farm located in the Heartland region; 0 Otherwise	0.17	(0.38)
*R_Northern Crescent*	1 if farm located in the Northern Crescent region; 0 Otherwise	0.16	(0.37)
*R_Northern Great Plains*	1 if farm located in the Northern Great Plains region; 0 Otherwise	0.06	(0.24)
*R_Prairie Gateway*	1 if farm located in the Prairie Gateway region; 0 Otherwise	0.11	(0.31)
*R_Eastern Upland*	1 if farm located in the Easter Upland region; 0 Otherwise	0.10	(0.30)
*R_Southern Seaboard*	1 if farm located in the Southern Seaboard region; 0 Otherwise	0.14	(0.34)
*R_Fruitful Rim*	1 if farm located in the Fruitful Rim region; 0 Otherwise	0.16	(0.37)
*R_Basin and Range*	1 if farm located in the Basin and Range region; 0 Otherwise	0.05	(0.22)
*R_Mississippi Portal*	1 if farm located in the Mississippi Portal region; 0 Otherwise	0.05	(0.22)
y2006	1 if data from year 2006; 0 Otherwise	0.35	(0.48)
y2007	1 if data from year 2007; 0 Otherwise	0.34	(0.47)
y2008	1 if data from year 2008; 0 Otherwise	0.32	(0.47)

Source: Agricultural Resource Management Survey (ARMS), 2006, 2007, and 2008.

The ARMS has a complex, stratified, multiframe design where observations in the ARMS represent a number of similar farms when using the provided expansion factors. The expansion factors are most useful and recommended when the full survey is used, generalizations about the entire population of farms is made based on the results, or simple univariate analysis is conducted. Under this scenario, the recommended method for calculating the variance is the delete-a-group jackknife procedure [20]. There is not clear or unanimous support for using the jackknife approach when using subsets of the data or complex, multivariate analyses. Goodwin and Mishra [21] argue that it is not clear whether stratification alters the likelihood function beyond the simple weights and whether it is appropriate to apply the predefined jackknife replicate weights to subsamples of the ARMS data. So, similar to El-Osta [22], we employ a bootstrapping technique rather than the jackknife procedure to remedy design problems in this subsample.

### Discussion of dependent and independent variables

The dependent variables in our off-farm labor supply equations are the hours per week worked off-farm by the operator and spouse, respectively. For each equation, we include explanatory variables for age, age squared, education, household size, distance from the off-farm job, off-farm wage, and whether they obtain health insurance from an off-farm source. Explanatory variables of the model are based on the theoretical model empirical literature on off-farm labor supply [3,16,23-25]. Refinements in the off-farm work literature has been extensive, largely using farm household cross-sectional micro data. The specific survey question asks respondents under the age of 65 whether they have insurance coverage from an off-farm job and 21% of the operators in our sample report that they were covered by an off-farm job. As expected, more spouses (30%) reported that they received insurance coverage from an off-farm employment source. As will be determined in the following section, we suspect that off-farm insurance coverage is jointly determined with the hours worked off-farm. Individuals working greater hours off-farm are more like to receive health insurance benefits and off-farm benefits are not likely to be received until a certain number of hours are accrued each week. The operator insurance and spouse insurance variables are found endogenous, so we will estimate the predicted probability of insurance coverage and include these estimates as explanatory variables in our labor supply equation.

**Figure 2:**
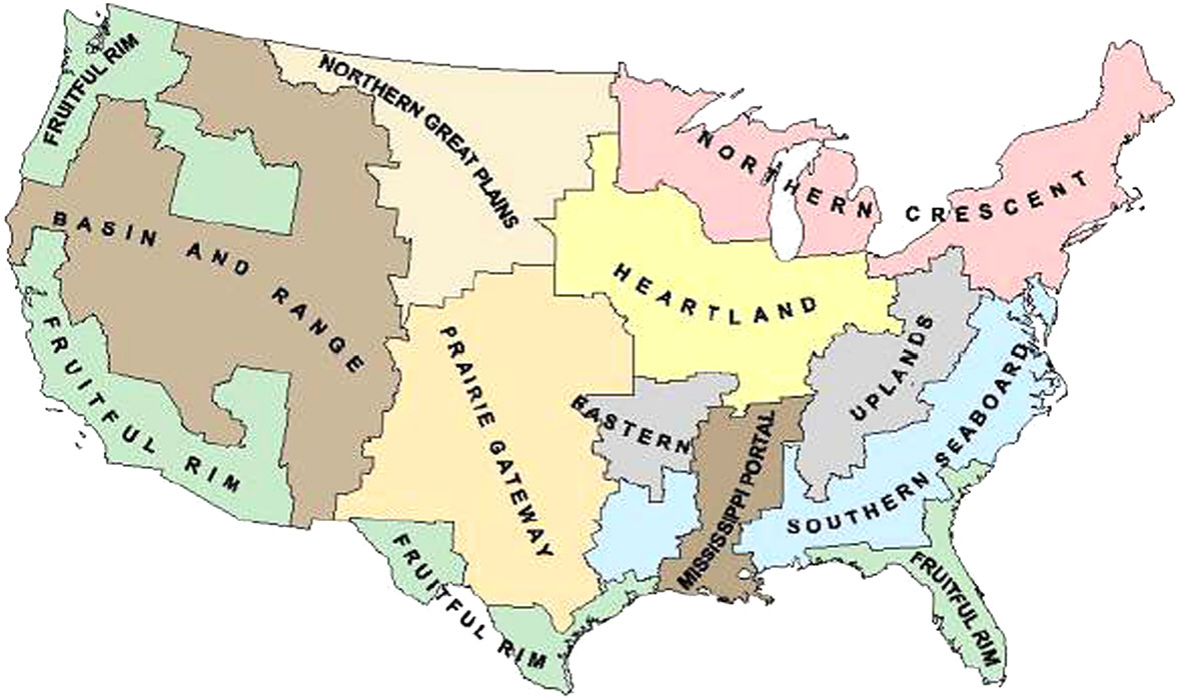
**Economic Research Service (ERS) Resource Regions.**

In addition to operator and spouse specific variables, we use farm, location, and time specific variables as explanatory variables. Farm-specific variables include decoupled and coupled government payments, total farm sales, and an indicator for dairy farms (which are specified due to the labor intensive nature of these farms). Previous studies that have included variables like coupled and decoupled payments, farm size^d^, specialization [2,16,17,21] and reported significant effects on off-farm labor supply of operators and spouses. We included location-specific variables such as metro/non-metro and ERS Resource region (Figure 2) as a proxy for local labor market conditions. Consistent with previous literature ([24]; Gunter [15,17,26]), we argue that inclusion of such variables captures the local labor market conditions, weather, growing crops and growing seasons which can significantly affect off-farm labor supply of operators and spouses. For the ERS Resource regions, the Mississippi Portal is used as the reference region in our study. Because we utilize a pooled sample, indicator variables to specify year are included. The reference year in this research is 2006.

## Results and discussion

Table 3 presents the results of dependence in dual employment using the Frank and Clayton copula functions, respectively. First, we consider the results of the symmetric Frank copula. We find evidence of a positive and highly significant relationship between the off-farm labor supply of farm operators and spouses. The correlation measure for this trial is consistent and ranges from 0.23 in 2006 to 0.19 in 2008. The pooled test of dependence is similar, measuring about 0.22. These estimates add to the confidence and power in using copulas to test for dependence. Finally, the dependence parameter estimates (column 3) are very consistent across the three surveys and pooled data as well. Findings from the Frank copula procedure guided our decision to use the Clayton copula to constrain the dependence parameter to (0, ∞).

**Table 3:** **Testing for dependence between hours worked off-farm by operator and spouse**

**Frank copula**	**Kendall’s τ**	**Dependence parameter**	**Standard error**
**Pooled**	0.2250	1.3844***	(0.0490)
**2008**	0.1896	1.1576***	(0.0858)
**2007**	0.2543	1.5760***	(0.0863)
**2006**	0.2280	1.4035***	(0.0827)
**Clayton copula**	**Kendall’s τ**	**Dependence parameter**	**Standard error**
**Pooled**	0.2744	0.7563***	(0.0230)
**2008**	0.2317	0.6030***	(0.0385)
**2007**	0.3135	0.9135***	(0.0426)
**2006**	0.2737	0.7536***	(0.0383)

*** indicates significance at the 1% level.

Results from the Clayton functional form are presented in the lower panel of Table 3. Again we find evidence of a positive and highly significant relationship between the off-farm labor supply of farm operators and spouses. Interestingly, the correlation correlations measured by the Clayton copula are larger. For example, the correlation measure ranges from 0.27 in 2006 to 0.23 in 2008. Finally, the dependence parameter estimates (column 3) are very consistent across the four surveys and pooled data as well. Findings here suggest that an increase in operators’ off-farm labor supply increases spouses’ off-farm labor supply; hence, we choose to model the impact of health insurance coverage on the off-farm labor supply of farm operators and spouses using a bivariate Tobit model rather than independent Tobit models.

The results in Tables 4 and 5 indicate a positive and significant relationship between the predicted probability of insurance coverage and the hours worked off-farm for both the operator and spouse. Jointly considering the results of our dependence estimates and insurance coverage variables, our empirical results support the theory that greater fringe benefits tend to increase the hours worked off the farm by both farm operators and spouses, *ceteris paribus.* Specifically, a one percent increase in the probability of having health insurance coverage from off-farm work increases off-farm hours by 1 and 0.8 hours per week for operators and spouses, respectively. Surprisingly, the hourly wage earned off-farm was not significant in either equation. This finding lends support to the relative importance of the fringe benefits component of the full off-farm wage.

**Table 4:** **Bivariate tobit results for hours worked off-farm by the operator**

**Variables**	**Parameter estimates**	**Standard error**	**Marginal effects**
*Op_Age*	−0.0777	(0.3219)	0.00000
*Op_AgeSq*	−0.0027	(0.0032)	0.00000
*Op_Educ*	0.1168	(0.2168)	0.00000
*Op_Miles*	0.1991^***^	(0.0107)	0.12894^***^
*Op_Hinsurance*	103.4670^***^	(3.2572)	99.4560^***^
*Of_Wage*	0.2044	(0.2896)	0.20439
*Decoupled_Payments*	−0.4075^***^	(0.0281)	−0.11850^***^
*Coupled_Payments*	−0.0333^**^	(0.0154)	−0.00946^**^
*Farm_Sales*	−0.0010^**^	(0.0004)	−0.00024^**^
*Household_Size*	0.9400^***^	(0.3191)	0.91646^***^
*Dairy_farm*	−28.5782^***^	(1.7309)	−12.6058^***^
*R_Heartland*	−0.4679	(2.1113)	−0.22681
*R_Northern Crescent*	2.1882	(2.2940)	1.20937
*R_Northern Great Plains*	−1.7238	(2.4179)	−0.83510
*R_Prairie Gateway*	3.2268	(2.1801)	1.76193
*R_Eastern Upland*	1.4987	(2.1677)	0.78834
*R_Southern Seaboard*	−5.6698^***^	(2.1646)	−2.45213^***^
*R_Fruitful Rim*	0.2730	(2.2709)	0.13857
*R_Basin and Range*	−1.6285	(0.9695)	−0.79391
*y2007*	−2.4322^**^	(0.9695)	
*y2008*	−3.5023^***^	(1.0426)	
*Constant*	−16.7046^*^	(9.9960)	
*Sigma Operator*	34.3934^***^	(0.4127)	
*Rho*	0.3652^***^	(0.0214)	
N		11,262	
*χ* ^*2*^		2,842.78^***^	

***, **, and * indicate significance at the 1%, 5%, and 10% levels, respectively.

**Table 5:** **Bivariate tobit results for hours worked off-farm by the spouse**

**Variables**	**Parameter estimates**	**Standard error**	**Marginal effects**
*Sp_Age*	0.8931^***^	(0.2571)	0.88983^***^
*Sp_Agesq*	−0.0115^***^	(0.0028)	−0.00004^***^
*Sp_Educ*	−0.5963^**^	(0.2479)	−0.00067^***^
*Sp_Miles*	0.0213^***^	(0.0024)	0.01651^***^
*Sp_Hinsurance*	85.2026^***^	(4.3399)	82.5941^***^
*Of_Wage*	0.1232	(0.1965)	0.00000
*Decoupled_Payments*	−0.0745^***^	(0.0134)	−0.02185^***^
*Coupled_Payments*	−0.0035	(0.0083)	−0.00110
*Farm_Sales*	−0.0012^***^	(0.0002)	−0.00028^***^
*Household_Size*	−0.5262^**^	(0.2428)	−0.01318^***^
*Dairy_farm*	−6.9291^***^	(0.9781)	−3.05645^***^
*R_Heartland*	0.9716	(1.3928)	0.52927
*R_Northern Crescent*	−1.3571	(1.5300)	−0.61083
*R_Northern Great Plains*	−1.7883	(1.5954)	−0.85499
*R_Prairie Gateway*	−2.0328	(1.4505)	−0.92525
*R_Eastern Upland*	−2.6948^*^	(1.4695)	−1.21925^*^
*R_Southern Seaboard*	−1.6355	(1.4328)	−0.73462
*R_Fruitful Rim*	−4.0687^***^	(1.5186)	−1.71412^***^
*R_Basin and Range*	−3.2057^*^	(1.7154)	−1.52402^*^
*y2007*	−1.2273^*^	(0.6605)	
*y2008*	−0.4432	(0.7134)	
*Constant*	−7.8118	(7.4883)	
*Sigma Spouse*	26.4324^***^	(0.2438)	
*Rho*	0.3652^***^	(0.0214)	
N		11,262	
*χ* ^*2*^		2,899.96^***^	

***, **, and * indicate significance at the 1%, 5%, and 10% levels, respectively.

Another variable of special interest in this study is decoupled payments. The coefficient on decoupled payments is negative and statistically significant at the 1 percent level of significance for both the operator and spouse. These results imply the number of hours worked off-farm by the operator and spouse will increase as funding for decoupled payments decline. With respect to coupled farm payments, decreased funding results in more hours worked off-farm but to a lesser degree than for decoupled payments. This relationship has been previously established in the literature with respect to the participation decision [3,17,27]. Whether the additional off-farm hours are drawn from farm labor or leisure is indeterminate from these results.

The demographic variables for the operator and spouse had different impacts on off-farm labor supply. The operator age, age squared, and education were all insignificant. Alternatively, the age of the spouse was found to increase the number of hours worked off-farm but at a decreasing rate. For example, results in Table 5 indicate that each additional year in the spouse’s age increases off-farm hours by about 0.90 hour per week. Household size also had a differential impact on the hours worked off-farm by the operator and spouse. Larger households were positively correlated with greater hours worked off-farm by the operator. Considering the documented relationship between income stability and off-farm labor supply [16], this makes sense: operators need a more stable and generous source of income to support larger families. However, larger households were found to be negatively correlated with hours worked off-farm by the spouse. This is likely due to increased value of at-home time and the established notion that spouses are increasingly responsible for household labor rather than monetary wages as household size grows [3,16,28].

In addition to operator and spouse characteristics, location and characteristics of the farm itself also play an important role in off-farm labor supply. Results in Tables 4 and 5 indicate that farms specializing in dairy production tend to have operators and spouses who work fewer hours off-farm. This result is expected due to the fact that dairy farming is more labor-intensive than many farming operations so operators and spouses allocate fewer (if any) hours for off-farm work [3,16]. Tables 4 and 5 also indicate that distance to the off-farm job has a positive and significant impact on the off-farm labor supply of farm operators and spouses. A plausible explanation may be that mileage traveled to off-farm work is considered as a fixed cost to the employee, and therefore greater travel distances require higher earnings—perhaps with fringe benefits—to justify the off-farm work trips (see [16]). Holding wages and all else constant, the worker must work greater hours off-farm to increase earnings—which may include fringe benefits.

Regional location of the farm is also an important factor in determining off-farm labor supply (demonstrated in Table 4 for the farm operator and Table 5 for the spouse). Farm operators located in the Southern Seaboard region (Figure 2) supply less off-farm labor (2.4 hours per week) compared to farms located in the Mississippi Portal region. This likely occurs because the Mississippi Portal region tends to have smaller farms specializing in livestock and mixed grains so operators of these farms are able to work more off the farm [2]. On the other hand, spouses of farming households located in the Fruitful Rim, Basin and Range, and Eastern Upland regions work less (1.2 to 1.7 hours per week) off the farm, compared to spouses in the Mississippi Portal region. With respect to the Fruitful Rim and Basin and Range, perhaps this result is due to the large number of fruit and vegetable farms in this area. Like dairy farms, the labor intensive nature of these farms may force spouses to devote more time to farm labor.

Dummy variables for the year in which the data was collected were included in the regression. Relative to 2006, both operators and spouses worked fewer hours off-farm in 2007. In 2008, operators but not spouses worked fewer hours off-farm than in 2006. This finding may suggest that economic conditions in the farm sector improved relative to the non-farm economy and thereby decreased off-farm labor supply of farm operators and spouses. Perhaps this is due in large part to the recession beginning in 2007. Finally, notice the estimate for the dependence parameter *rho* (0.3652). The positive and significant correlation between error terms confirms the findings of our copula test.

## Conclusions

Previous research on off-farm labor supply has ignored the role of employer-sponsored health insurance coverage in determining farm operator and spouses’ decisions to work off the farm. Nearly 40 percent of individuals from farm households purchase health insurance coverage directly from the vendors, compared to 7 percent of all other U.S. households. This study provides evidence that observed off-farm labor participation of farm operators and spouses is indeed influenced by employer-sponsored health care coverage. We find that government payments, whether coupled or decoupled, have a negative effect on the off-farm labor supply of farm operators and spouses. Our study also demonstrates a strong positive relationship between the probability of health insurance coverage and the off-farm labor supply by the operator and spouse. In addition, human capital, farm characteristics, and participation in government programs are significant determinants of off-farm labor supply by the operator and spouse. We also find characteristics of the family and spouse play an important role on the spouse’s decision to work off the farm.

Recent changes in both healthcare and agricultural policy may have a negative impact on the hours worked off-farm by farm households. The 2014 Farm Bill eliminated decoupled payments and instead expanded risk management tools available to farmers. Based on our results, lower decoupled payments over the duration of the farm legislation would lead to fewer hours worked away from the farm. Lower income from eliminating decoupled payments and fewer hours worked off farm result in lower levels of income, so the expectation is that the farm household works more hours on the farm rather than increasing leisure.

Similarly, this shift toward less off farm work and greater hours of farm labor may be furthered by healthcare policy. As part of the Affordable Care Act (ACA), insurance exchanges were created that allow very small business owners access to health care and lower expected prices. This change decreases the relative total wage differential between farm and off farm labor and is expected to lead to fewer hours worked off farm. This is especially true if the operator or spouse receives non-pecuniary benefits from farming.

Drawing from these conclusions, a consideration worthy of additional research is the impact on farm risk from these changes. This literature has shown that stable income sources like decoupled payments and off-farm labor are important risk management tools that help stabilize the annual income of farmers. With lower decoupled payments and resulting decrease in off-farm work, does the expansion of risk management programs in 2014 Farm Bill sufficiently offset and further reduce the risk of farming?

## Endnotes

^a^Off-farm provisions have been largely responsible for: (1) closing the income gap between farm and nonfarm households [2]; (2) food consumption and nutrition [27,29]; and (3) farm input usage [30].

^b^El-Osta, Mishra and Ahearn [17] found that whether one used estimated payments or actual payments, the qualitative results did not differ. Hence, we use actual decoupled and coupled payments in our model.

^c^Ideally, inclusion of operator/spouse’s health uncertainty in theoretical model serves as the foundation of health insurance. However, we have not included uncertainty impacts in this simple framework.

^d^Generally, farm size and farm asset values are positively correlated. We include farm size (total farm sales) as an indicator of financial variable and also wealth effect on off-farm labor participation. Families with large farm assets values are less likely to work of the farm.
